# Posterior Intraparietal Sulcus Mediates Detection of Salient Stimuli Outside the Endogenous Focus of Attention

**DOI:** 10.1093/cercor/bhab299

**Published:** 2021-08-31

**Authors:** Tarik Jamoulle, Qian Ran, Karen Meersmans, Jolien Schaeverbeke, Patrick Dupont, Rik Vandenberghe

**Affiliations:** Laboratory for Cognitive Neurology, Department of Neurosciences, KU Leuven, Leuven, Belgium; Laboratory for Cognitive Neurology, Department of Neurosciences, KU Leuven, Leuven, Belgium; Laboratory for Cognitive Neurology, Department of Neurosciences, KU Leuven, Leuven, Belgium; Laboratory for Cognitive Neurology, Department of Neurosciences, KU Leuven, Leuven, Belgium; Laboratory for Cognitive Neurology, Department of Neurosciences, KU Leuven, Leuven, Belgium; Laboratory for Cognitive Neurology, Department of Neurosciences, KU Leuven, Leuven, Belgium; Neurology Department, University Hospitals Leuven, Leuven, Belgium

**Keywords:** cytoarchitectonics, endogenous selection, exogenous capture, intraparietal sulcus, visual attention

## Abstract

Visual consciousness is shaped by the interplay between endogenous selection and exogenous capture. If stimulus saliency is aligned with a subject’s attentional priorities, endogenous selection will be facilitated. In case of a misalignment, endogenous selection may be compromised as attentional capture is a strong and automatic process. We manipulated task-congruent versus -incongruent saliency in a functional magnetic resonance imaging change-detection task and analyzed brain activity patterns in the cortex surrounding the intraparietal sulcus (IPS) within the Julich-Brain probabilistic cytoarchitectonic mapping reference frame. We predicted that exogenous effects would be seen mainly in the posterior regions of the IPS (hIP4–hIP7–hIP8), whereas a conflict between endogenous and exogenous orienting would elicit activity from more anterior cytoarchitectonic areas (hIP1–hIP2–hIP3). Contrary to our hypothesis, a conflict between endogenous and exogenous orienting had an effect early in the IPS (mainly in hIP7 and hIP8). This is strong evidence for an endogenous component in hIP7/8 responses to salient stimuli beyond effects of attentional bottom-up sweep. Our results suggest that hIP7 and hIP8 are implicated in the individuation of attended locations based on saliency as well as endogenous instructions.

## Introduction

Attentional orienting is typically separated into 2 types: Endogenous orienting is the process behind the voluntary, top-down control of the allocation of attention; Exogenous orienting refers to the bottom-up automatic capture of attention by stimuli with a high visual salience, for example, based on high contrast or sudden onset (Parkhurst et al. 2002). If stimulus saliency is aligned with a subject’s attentional priorities, selection will be facilitated. In case of a misalignment, endogenous selection may be compromised (automaticity of attentional capture; Pashler 1999).

Visual salience and behavioral relevance have been thoroughly researched separately (Somers et al. 1999; Buracas and Boynton 2007; Serences and Yantis 2007; Murray 2008; Geng and Mangun 2009; Zhang et al. 2012; Sprague and Serences 2013). Other neuroimaging studies have manipulated saliency and relevance orthogonally (Melloni et al. 2012; Bertleff et al. 2016; Sprague et al. 2018). These studies found that saliency maps are hierarchically organized in early visual areas (V1–hV4). Although representation of endogenous control can be found as early as V2, it dominates in parietal and frontal areas (namely the intraparietal sulcus [IPS] and the frontal eye fields [FEF]). Exogenous capture has a very strong effect, but it has been shown that endogenous orienting can successfully inhibit it through increases in the neural response to the attended stimulus in medial parietal regions (Bertleff et al. 2016). Recent findings suggest that activation profiles in IPS0 (a posterior retinotopic area of IPS) only index the location of relevant objects, even if irrelevant but salient stimuli are present in the visual scene (Sprague et al. 2018). Meyer et al. (2018) have shown that parietal activity is higher than frontal activity for exogenous orienting, whereas frontal activity is higher than parietal activity for endogenous orienting. Whether the IPS functional activity is more related to endogenous or exogenous orienting is an ongoing debate (Vandenberghe et al. 2005; Serences et al. 2005; Corbetta et al. 2008; Vandenberghe and Gillebert 2009). According to one mainstream model of visual attention, the middle IPS is mostly involved in endogenous orienting while other regions such as the temporoparietal junction (TPJ) mostly mediate exogenous orienting (Corbetta et al. 2008), but there is evidence in the neuroimaging literature that IPS plays an important role in both types of attention (Woldorff et al. 2004; Kincade et al. 2005; Vandenberghe et al. 2005; Serences et al. 2005; Corbetta et al. 2008; Mevorach et al. 2008; Geng and Mangun 2009; Vandenberghe and Gillebert 2009; Cieslik et al. 2010; Vandenberghe and Gillebert, 2013).

Recent advances in probabilistic mapping of postmortem brains show as many as 8 different cytoarchitectonically distinct areas within the human IPS (named hIP1–hIP8) (Scheperjans et al. 2008a, 2008b; Richter et al. 2019). The cytoarchitectonic reference frame provides a parcellation that is easily reproducible between centers and provides a common neuroanatomical framework. That framework is advantageous for analyzing and reporting functional responses in neuroanatomically complex structures such as the IPS. hIP8, hIP7, and hIP3 are located in the medial wall of IPS, whereas hIP4, hIP5 and hIP6, hIP2, and hIP1 are located in the lateral wall of IPS (Richter et al. 2019). The present study applied this cytoarchitectonic reference frame. Clusters of IPS areas have been suggested, grouping the posterior visuotopic areas hIP7 and hIP4 as a caudo-medial cluster and the anterior visuotopic areas hIP8, hIP5, and hIP6 as a rostro-lateral cluster. Areas hP01, hIP7, hIP4 (Richter et al. 2019), and hIP3 (Uddin et al. 2010) have stronger bilateral connectivity with extrastriate visual and occipito-temporal cortex, whereas areas hIP8, hIP5, hIP6 (Richter et al. 2019), as well as hIP1 and hIP2 (Uddin et al. 2010) are more strongly connected with insula, premotor, frontal opercular, and prefrontal cortex. Inferences about the function of these areas are principally based on metadata derived from the Brainmap database, rather than direct contrasts between conditions within a same experiment within individuals (Laird et al. 2009). These functions vary widely and also overlap between areas (Richter et al. 2019). Direct comparisons of the function of these areas within a same experiment have rarely been performed. Gillebert et al. (2013) directly contrasted a spatial cueing paradigm with an attentional selection paradigm and found that area hIP3 was mostly involved in selection between competing stimuli compared with attentional reorienting. Reorienting following an invalid cue relied on right area PF, an inferior parietal area at the TPJ (Gillebert et al. 2013). Neuroanatomic overlay with visuotopic maps, derived from memory saccade paradigm, indicates that hIP7 and partly also hIP4 may correspond to what has been called IPS0 (Wang et al. 2015; Richter et al. 2019). hIP7 has cytoarchitectonic features resembling those seen in extrastriate cortex (Richter et al. 2019). IPS1 colocalized mostly with hIP8 (Richter et al. 2019). In addition to the 8 hIP areas, other cytoarchitectonic parietal areas of interest in this study include the medial superior parietal areas 7A and 7P and the anterior part and the posterior part of area PG (PGa and PGp, respectively) in the angular gyrus.

Based on prior studies, we were mainly interested in how the different cytoarchitectonic areas of the IPS process salient stimuli depending on whether or not they fall within the locus of endogenous orienting (termed saliency-congruent versus -incongruent in the remainder of the paper). We predicted that in posterior cytoarchitectonic areas such as hIP4, hIP7, and hIP8, mainly effects of saliency would be seen, whereas in more anterior cytoarchitectonic areas of the IPS (hIP1–hIP3) the cognitive control demands elicited by the response conflict between exogenous and endogenous orienting would lead to higher activity. We examined this hypothesis within a cytoarchitectonic reference frame, encompassing the cytoarchitectonic areas within the medial and lateral wall of IPS as well as the adjacent inferior and medial superior parietal areas. To our knowledge, no studies have manipulated the spatial distribution of targets over the visual field, in a multi target environment, whereas manipulating visual salience and behavioral relevance orthogonally within the neuroanatomical framework of cytoarchitectonic maps, using both uni- and multivariate analysis.

## Material and Methods

### Participants

In total, 26 university students (median age = 25, range: 19–28 years old, 19 women and 7 men) participated. The sample size is in line with previous studies using univariate or multivariate analyses. A sample size of 26 allows for detection of reproducible pattern changes in healthy young adults (Gillebert et al. 2012; Bruffaerts et al. 2013; Liuzzi et al. 2015, 2017, 2019; Neyens et al. 2017; Meersmans et al. 2020). Subjects were all native Dutch speakers and right-handed. All subjects were healthy and had no psychiatric or neurological history. All subjects first took part in a behavioral experiment, 24 (median age = 25, range: 19–28 years old, 6 men) out of the 26 subjects participated next in a functional magnetic resonance imaging (fMRI) experiment (2 subjects refused participating). Out of the 24 subjects, 2 had to be removed prior to data analysis because of excessive head movement. All participants gave written informed consent in accordance with the Declaration of Helsinki. The study was approved by the Ethics Committee of the University Hospitals Leuven.

### Experimental Design

Participants performed a change-detection task (Gillebert et al. 2013). In both the behavioral and the fMRI experiment, an array of 8 stimuli was presented on an imaginary circle at 3.5° eccentricity. In half of the runs, the array consisted of 2 letters and 6 numbers and, in half, of 2 numbers and 6 letters. At the beginning of each run, participants were informed which type of target (letter or number) they had to attend to. Targets were always the minority stimuli (the 2 letters or the 2 numbers, respectively). The target selection criterion (letter or number) was counterbalanced over runs within each individual. The selection criterion stayed the same throughout a given run. The array was followed by a visual mask, a delay, and a probe phase where a central stimulus was presented. Subjects were instructed to fixate the central fixation point and to respond as fast as possible whether the identity of the probe matched the identity of either of the 2 targets. The letters were selected randomly from a set of 18 consonants (BCDFGHJKLMPQRSTVXZ) and the numbers from 1 to 9. No letter or number could be present twice within a given array. Targets and distracters (size 0.75°) were centered around a central fixation dot (diameter 0.25°) on which subjects had to maintain fixation. Stimuli were separated by an angle of 45° starting from 0° at the *X*-axis.

In an orthogonal manner, we manipulated exogenous orienting by manipulating the color of the stimuli. In each display there were 2 red stimuli and 6 blue stimuli or vice versa. The 2 colors were matched in luminance with a LS-100 Luminance Meter (15.2 cd/m^2^; Blue RGB [68,68,255]; and Red RGB [255,5,5]). The probe had no specific color (white). The color of the targets and distracters were irrelevant for the goal of the task. Subjects were aware of that fact and were instructed to ignore the color.

**Figure 1:**
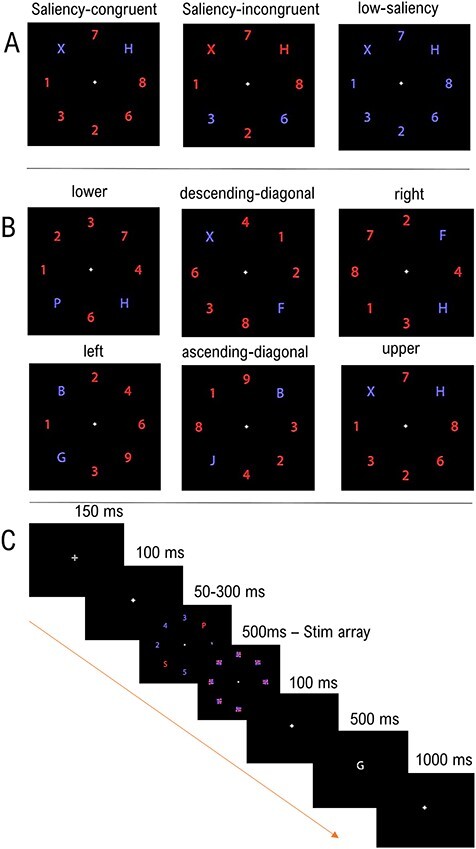
(*A*) The 3 possible saliency conditions in the experimental design. On the leftmost panel, the 2 letter targets are of a different color than the distracters and saliency is congruent with task-relevance. In the middle panel, in the saliency-incongruent condition, the 2 target pairs are in red alongside 4 other distracters, whereas the 2 opposite distracters to the target pairs are in the other color. Hence, saliency is incongruent with task-relevance. In the righmost panel, in the low-saliency condition, all stimuli share the same color. (*B*) The 6 squares depict the 6 possible spatial configurations for the 2 targets, presented here for the saliency-congruent condition. (*C*) Example trial of the paradigm. In this trial, the focus of exogenous and endogenous attention coincided, saliency is thus congruent with task-relevance.

Stimulus salience was manipulated over 3 conditions (Fig. 1*A*). In the saliency-congruent condition, the 2 targets were of a same color and all distracters of the other color. Hence, the spatial focus of endogenous and exogenous attention coincided with the location of the targets. In the saliency-incongruent condition, 2 of the 6 distracters were of one color, and the remaining stimuli of the other color, including the 2 targets. Hence, there was a spatial dissociation between the location of the salient stimuli and that of the targets. Finally, in the low-saliency condition, all stimuli were of a same color. The 2 colors were counterbalanced within each run so that in each saliency condition, the 2 targets appeared equally often in blue or in red. The trial order for the salient color was pseudorandomized, with as constraint that the same color could not be used more than twice in a row.

All possible combinations of 2 targets and 6 distracters over the 8 locations would have amounted to 28 combinations of spatial target-distracter configuration. In order to reduce the number of possible combinations and increase the power to detect differences between configurations, we restricted the possible locations of targets to the middle position of each visual quadrant (along the diagonal at an angle of 45° with respect to each major axis). This led to 6 possible target configurations over the 4 visual quadrants: The 2 targets could appear in the 1) upper visual field; 2) lower visual field; 3) left visual field; 4) right visual field; 5) on the ascending diagonal (lower left and upper right visual quadrant); and 6) or on the descending diagonal (upper left and lower right visual quadrant; Fig. 1*B*). The location where the 2 salient stimuli appeared was also restricted to these 6 configurations. In the saliency-congruent condition, the 2 salient stimuli coincided with the 2 targets. In the saliency-incongruent condition, the 2 salient stimuli appeared in the spatial configuration opposite to the 2 targets, that is, when the 2 targets were in the upper location, the 2 salient stimuli were in the lower location, and so on. This restriction was applied in order to keep the positions between salient congruent and incongruent targets/distracters matched. As relevant (or irrelevant-salient) could only appear on 4 locations out of the 8 on the screen, relevant non-salient stimuli had to appear in the mirrored position to the irrelevant salient ones. Participants were not made aware explicitly that the targets could only appear at 4 locations. This was assessed by a simple question at the end of the experiment. Gaze fixation was monitored using Eyelink 1000.

At the start of each experiment, the instructions were presented on the screen. Before the start of each run, text on the screen indicated whether the targets for this run were letters or numbers. After an initial warning cue (150 ms) consisting of an enlarged fixation dot (diameter 0.45°) and a 100-ms delay, the stimulus array was displayed for a period of 50–300 ms (Fig. 1*C*). The duration of the array for each saliency condition was titrated per subject in a prior behavioral experiment to equate performance accuracy between conditions during the fMRI experiment. The display was immediately followed by a mask of variable length. Each stimulus position was occupied by a patterned mask (size 0.75°) consisting of jumbled letter features in the red and blue colors used in the stimulus display (Vangkilde et al. 2011; Gillebert et al. 2012; Phillips 1974). The total duration of the stimulus and mask display was held constant at 500 ms. After a 100-ms delay, a central probe stimulus was presented for 500 ms and subjects had to indicate by key press whether the probe matched either of the 2 targets. The response window was fixed at 2000 ms. In half of the trials, the probe was identical to 1 of the 2 targets, and in half it was different. Match/mismatch of a given trial was pseudo-randomized in a 3-back manner such that repetition of a mismatch trial was limited to 2 in a row, and was counterbalanced across all 3 conditions.

### Prior Behavioral Experiment

As saliency is known to facilitate attentional capture (Pashler 1999), we expected differences in subjects’ performance based on the experimental condition. The purpose of the behavioral experiment was to determine the array duration parameters that would allow to match the accuracies of participants between the different conditions during the fMRI experiment. The total duration of array plus mask was always held constant, the relative duration of array versus mask was adapted in order to equate performance between conditions.

Subjects were tested in a dimly lit experiment room on a 120-Hz computer screen. The experiment was displayed and recorded with MATLAB and Psychtoolbox (Brainard 1997). Participants responded by pressing either of 2 keys (“J” and “K”) with their index and middle finger on a standard keyboard which were identified with colored tape. To control for laterality of the response, for half of the participant, “J” indicated a “yes” response and “K” a “no” response, and inversely for the other half. The stimulus duration was modified online with a 3-down 1-up staircase procedure. Initial duration of the stimulus array was 300 ms, which could be reduced by 16 ms after 3 good answers in a row or lengthened by 16 ms after 1 wrong answer. The duration of the mask display was modified accordingly to ensure a total length of 500 ms for the combined duration of the stimulus array and the mask. The behavioral experiment was divided in 6 runs of 144 trials each. All 6 spatial configurations were presented 24 times per run. In the behavioral experiment, all participants started with 2 runs of the saliency-congruent condition, followed by 2 runs of the low-saliency condition, and finally 2 runs of the saliency-incongruent condition. This particular design was specific to the behavioral experiment. Participants were allowed to take a break of 2 min after each run. Half of the participants started the first run with letters as target, and inversely for the other half. For each subject and for each condition, we computed the median duration of the stimulus array in the last half of each run, in which a given condition appeared. This value was then used as the fixed stimulus array duration in the fMRI experiment.

### fMRI Experiment

Participants were asked to return on a separate day for the fMRI session, where we examined how brain activity differed between conditions. The total duration of array plus mask was held constant, whereas the duration of the stimulus array varied as a function of the values obtained from the behavioral session to equate performance. Participants responded by pressing 2 buttons from an MRI-compatible response box with their index and middle finger. For each subject, the laterality of “yes” and “no” response was identical to the one during the behavior experiment. The fMRI experiment was divided in 6 runs of 144 trials each: 36 trials for each of the 3 conditions and 36 null trials. During the null trial, only a stable fixation cross was presented for 3.25 s. Each trial lasted 3.25 s (Fig. 1). For each condition, a given spatial configuration was presented 6 times, totaling 18 repetitions across conditions in a run. Trials were randomized in bins of 4 for each of the saliency-condition plus the null condition, and all possible combinations of the 4-event types were equally likely (3-back balance). For the spatial configuration, the only restriction applied was that no target configuration could appear more than twice in a row. The relatively short intertrial interval (3.25 s) and the fact that trials were closely spaced together could lead to overlap in the resulting hemodynamic response in the fMRI. Proper deconvolution of the hemodynamic response can be achieved when carefully randomizing the sequence of event-type (3-back balance in our case; Dale and Buckner 1997).

### Analysis of the Behavioral Data

In the behavior experiment, the effect of the saliency condition on subject’s performance was tested with one-way repeated measures analysis of variance (ANOVA) with each saliency condition as factor. Post-hoc pairwise comparisons were performed with a Tukey’s honest significant difference (HSD) test. In the fMRI experiment, we investigated the effect of saliency on subject’s performance (accuracy and reaction times) by means of a repeated measures ANOVA with saliency (3 levels: saliency-congruent, saliency-incongruent, and low-saliency) as within-subject factor.

### MRI Acquisition and Preprocessing

Structural and functional images were acquired on a 3T Philips Achieva system equipped with a 32-channel head volume coil. Participants first underwent a structural scan consisting of a T1-weighted 3D turbo-field-echo sequence (repetition time [TR] = 6.71 ms, echo time [TE] = 3.09 ms, In-plane resolution = 0.97 mm, and slice thickness = 1.2 mm). Functional images were obtained with a T2^*^ echoplanar image sequence consisting of 51 transverse slices (TR = 1000 ms, echo time = 32 ms, voxel size = 2.75 × 2.75 mm^2^, slice thickness = 3.75 mm, and Sensitivity Encoding [SENSE] factor = 2), with a field of view (220 × 127.5 × 200 mm^3^) covering the entire brain. Each run was preceded by 4 dummy scans to allow for saturation of the blood oxygen level-dependent (BOLD) signal.

Images were preprocessed and analyzed with Statistical Parametric Mapping SPM12 (Wellcome Trust for Neuroimaging, University College London). Scans were first realigned and slice time corrected, then co-registered to the anatomical T1 image. The anatomical T1 image was segmented in white matter, CSF, and gray matter. Using the warping parameters from the segmentation of the T1 image, the functional images were normalized into Montreal Neurological Institute (MNI) space and resliced to a voxel size of 3 × 3 × 3 mm^3^. The T1 image was normalized as well and resliced to a voxel size of 1 × 1 × 1 mm^3^. Functional images were smoothed using a Gaussian filter with a kernel size of 6 × 6 × 6 mm^3^. Unsmoothed images were used for the multivariate pattern analysis (MVPA).

### ROI-Based Analysis

#### Regions of Interest

Thirteen cytoarchitectonic regions were selected a priori for region-of-interest (ROI)-based analyses (Fig. 4). These regions were extracted from the Julich-Brain brain atlas with the SPM anatomy toolbox (v3.0; Eickhoff et al. 2005, 2006, 2007). In this probabilistic atlas, each voxel is given a probability that represents the number of postmortem brains in whom the voxels belong to a cytoarchitectonic area divided by the total number of brains examined. The anatomy toolbox allows one to extract “maximum probability map,” in which each voxel is attached to the cytoarchitectonic area for which it has the highest probability (see Supplementary Material). In order to define these regions functionally, we constructed ROIs based on those 12 cytoarchitectonic areas (Caspers et al. 2006, 2008; Richter et al. 2019). We used the same inclusion criteria as defined by Gillebert et al. (2012)—for each ROI, we selected the maximum probability threshold for which the ROI size was larger than 29 voxels (783 m^3^). The probability threshold was lowered by 10% increment until this criterion was met (see Table 1). This method results in a set of ROIs that are relatively similar in size, with voxels that represent the core of the probabilistic map corresponding to the cytoarchitectonic area (see Supplementary Fig. 1; Gillebert et al. 2012). Next, a subject-specific ROI was created by overlaying the resulting ROIs with the subjects’ gray matter mask that was obtained after the segmentation of the anatomical T1 image (threshold = 0.3; Adamczuk et al. 2013). For group analyses, a composite gray matter mask over all subjects was created by summing all bias-corrected scans in MNI space.

**Table 1:** *P*-values of the one-sample *t*-test on the accuracy of the SVM classifier between the 3 different saliency-conditions: saliency-congruent versus saliency-incongruent, saliency-incongruent versus low-saliency, and saliency-congruent versus low-saliency

Region	Comparison	Accuracy	95% CI	*P*
hIP7	SI vs. SC	57.4	(51.5; 63.3)	**0.004**
	SI vs. LS	58.4	(52.5; 64.3)	**0.002**
	SC vs. LS	50.5	(44.7; 56.4)	0.430
hIP8	SI vs. SC	55.7	(49.8; 61.6)	*0.018*
	SI vs. LS	59.9	(54.0; 65.8)	**0.002**
	SC vs. LS	52.7	(46.8; 58.5)	0.174
hIP4	SI vs. SC	56.9	(51; 62.8)	*0.007*
	SI vs. LS	53.9	(48; 59.8)	*0.045*
	SC vs. LS	56.7	(50.8; 62.6)	**0.004**
PGa	SI vs. SC	50.9	(45; 56.8)	*0.018*
	SI vs. LS	3.22	(48.9; 60.6)	*0.007*
	SC vs. LS	55	(49.1; 60.9)	**0.002**

*Notes:* Bold *P*-values indicated significant effect after correcting for 12 multiple comparisons. Only areas where a significant effect was present after correction are shown here. For a complete overview of the cytoarchitectonic areas tested we refer to Supplementary Table 2. *Abbreviations:* vs. = versus; SC = saliency-congruent; SI = saliency-incongruent; LS = low saliency.

#### ROI Based Multivariate Analysis

First, we used multivariate pattern classification analysis (MVPA), where we trained and tested a classifier to discriminate between the 3-event types (saliency-congruent, saliency-incongruent, and low-saliency) based on the activity pattern in each of the 12 Jülich cytoarchitectonic areas. Pattern decoding was executed within the MATLAB environment with SPM12 and the pattern classification tools available in The Decoding Toolbox (Hebart et al. 2015). For each decoding analysis, a general linear model (GLM) with conditions of interest was created using unsmoothed images. Parameter estimates (β maps) were extracted for each of the 3-event types (saliency-congruent, saliency-incongruent, and low-saliency) for each subject. For each condition, a β map specific vector was derived and used as input for the Support Vector Machine (SVM). All classification analyses followed a leave-one-out 6-fold cross-validation procedure: Five runs were used for training and 1 for testing. The classification significance of the averaged accuracy was assessed using a one-sample *t*-test. All ROI-based analyses were applied on the 12 ROIs. For each analysis, a GLM was created with SPM using unsmoothed functional images. We first examined how accurately the SVM could classify between the 3-event types. The GLM contained 3 regressors corresponding to each saliency condition and 6 motion regressors. All trials were included. The SVM model thus contained 3 classes (saliency-congruent, saliency-incongruent, and low-saliency) that were compared in a one-versus-one manner, totaling 3 comparisons.

In a second analysis, we focused on the possible difference in brain patterns between the location of targets over the visual field. We applied SVM classification between pairs of non-overlapping target locations. The GLM model contained 6 regressors representing the 6 possible spatial configurations of the target pair over the stimulus array, and 6 motion regressors. The target pair could be positioned in the 2 left visual quadrants (left-pairs), the 2 right visual quadrants (right-pairs), the 2 upper quadrants (upper-pairs), the 2 lower quadrants (lower-pairs), or diagonally in the upper left quadrant and the lower right quadrant and inversely (diagonal-pairs). The analysis was restricted to the saliency-congruent condition as the location of attention can be most reliably estimated under these circumstances. The SVM analysis was applied between left versus right target pairs.

#### ROI-Based Univariate Analysis

Next, we performed a region of interest analysis on the 12 Julich cytoarchitectonic ROIs of prior interest to examine possible differences between the conditions. For each region of interest, we derived the fMRI response pattern by calculating the area under the curve of the BOLD response within every voxel between 2 and 8 s after stimulus onset per subject and per condition (Bruffaerts et al. 2013). To study the possible interaction between regions, the BOLD response of each ROI for each condition was normalized to the mean over all-conditions within each ROI to further reduce any effect that inter-regional differences in overall response amplitude could have. This was done by subtracting the response of each saliency-condition to the mean of each ROI. The area under the curve of the resulting responses were then computed and put in a one-way repeated measure ANOVA with the 3 event-types as factors (saliency-congruent, saliency-incongruent, and low-saliency). The following contrasts were then evaluated between each ROI using paired-sample *t*-tests: 1) saliency-incongruent minus saliency-congruent, 2) saliency-incongruent minus low-saliency, and 3) saliency-congruent minus low-saliency. Bonferroni correction for multiple comparisons was applied (α = 0.0042).

### Whole-Brain Analysis

#### Whole-Brain Multivariate Analysis

In order to evaluate any effects occurring outside the regions of a priori interest, we performed a searchlight analysis that was complementary to the ROI-based analysis. We searched for activity patterns throughout the brain where the classifier could distinguish between the 3 saliency conditions. For this SVM searchlight, we used the saliency GLM model with the 3 conditions as regressors. We used a spherical searchlight with 12-mm radius in all gray matter voxels (with a gray matter probability threshold set at 0.3), resulting in voxel-wise map of classification accuracies per subject. These maps were smoothed using a Gaussian kernel (kernel size of 6 × 6 × 6 mm^3^) and used in a random effects analysis. Significance of the classification accuracy was determined with one-sided *t-*tests (uncorrected voxel-level *P* < 0.001 and FWE-corrected cluster-level *P* < 0.05). In order to control for interindividual differences in stimulus array duration or response-time, we used both measures as regressors in the second-level *t*-test for all 3 comparisons. We first calculated the relative difference in array duration between all-conditions. We calculated this relative difference per subject by taking the absolute difference between 2 conditions divided by the absolute value of their arithmetic mean (|*x* − *y*|/([*x* + *y*]/2). The relative difference in array duration and RTs were added as a covariates in the multivariate searchlight analysis. Relative differences per subject were regressed out during the second level SPM analysis.

#### Whole-Brain Univariate Analysis

Finally, in order to study the voxelwise differences in response amplitudes between the different conditions, a classical whole-brain univariate analysis was performed with SPM. Smoothed images from the fMRI experiment were analyzed using a random effects GLM with 4 regressors corresponding to each of the conditions plus the null condition and 6 motion regressors. All trials were included in the analysis. Specific contrasts were estimated per subject using one-sample *t*-test following a priori comparisons: 1) saliency-incongruent > saliency-congruent and the inverse; 2) saliency-incongruent > low-saliency and the inverse; and 3) all-conditions > baseline (null condition). The resulting contrast images were analyzed in a second-level random effects analysis. For all contrasts, the significance level was set at a cluster-level *P* < 0.05 FWE corrected for the whole brain, with the voxel-level threshold set at uncorrected *P* < 0.001. As in the multivariate analysis, we used interindividual differences in stumulus array duration and response times as covariates.

## Results

### Behavioral Analysis

#### Behavioral Experiment

**Figure 2:**
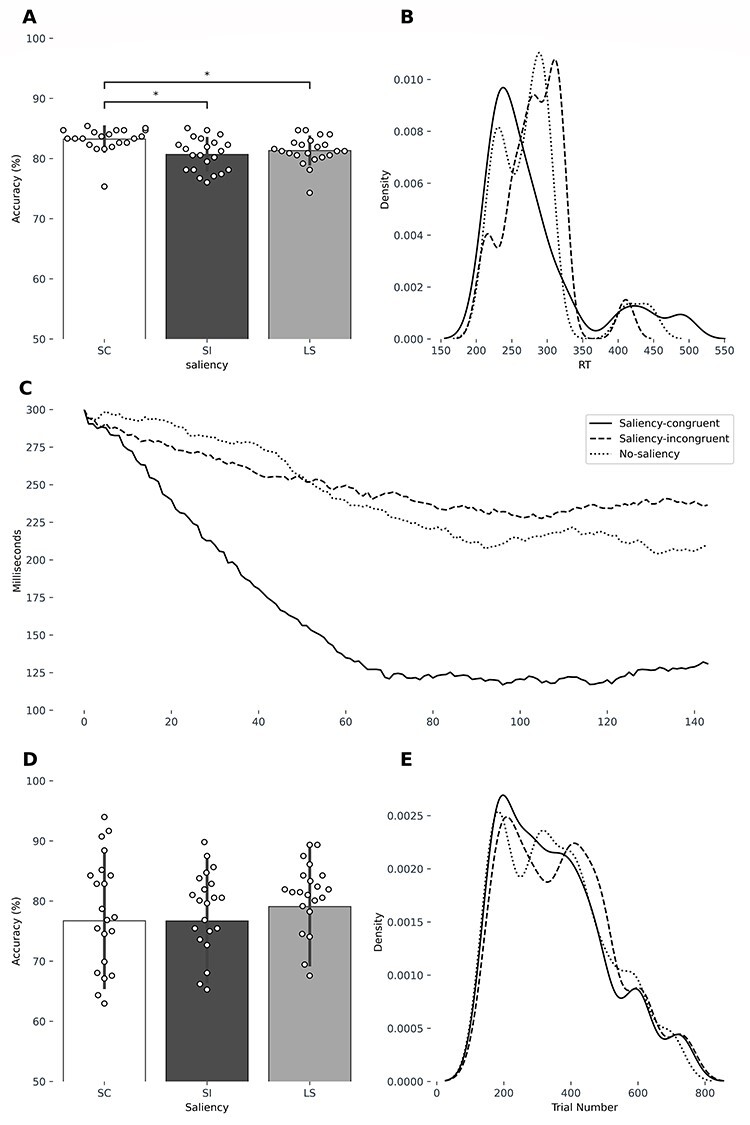
Behavioral experiment. (*A*) Mean accuracy in the behavioral experiment over all subjects across the 3 saliency-condition. (*B*) Reaction time kernel density distribution for the 3 saliency conditions. The 3 conditions did not statistically differ in terms of reaction time. (*C*) Evolution of the duration of the stimulus array during the staircase procedure. The duration of the stimulus array has been averaged over all subjects. The *x*-axis shows the trial order from the first to the last (144 trials total). The *y*-axis shows the duration of the stimulus array, starting from 300 ms to the minimum duration reached by the subjects. fMRI experiment: (*D*) Mean accuracy scores in the fMRI experiment over all subjects across the 3 saliency-condition and the 3 target axis configurations. SC = saliency-congruent, SI = saliency-incongruent, and NS = low-saliency. (*E*) Reaction time kernel density distribution and for the 3 saliency conditions.

One-way repeated measures ANOVA of performance during the behavioral experiment (applied on all trials presented during the staircase procedure) showed a main difference of accuracy between the 3 conditions (Fig. 2*A*; *F*_2,42_ = 9.91, *P =* 0.003). Post-hoc comparisons showed that accuracy in the saliency-congruent condition was significantly higher than in the saliency-incongruent condition (*t =* 3.476, *pcorr =* 0.0067) and than in the low-saliency condition (*t =* 2.918, *pcorr =* 0.024). There were no differences in accuracy between the saliency-incongruent and the low-saliency condition (Fig. 2*B*). The accuracy differed also when selecting only trials after the plateau (taken as the middle of the run) had been reached, (*F*_2,42_ = 6.07, *P* = 0.0048), Post-hoc comparison showed that saliency-congruent trials had a higher accuracy than low-saliency (*t* = 2.24; *P* = 0.03) trials and saliency-incongruent trials (*t* = 2.02, *P* = 0.05). Although the 2 sensorially matched conditions (saliency-congruent and saliency-incongruent) showed a clear difference in performance, the low-saliency condition was as difficult as the saliency-incongruent condition. Thus, although saliency-congruent and saliency-incongruent are the closest conditions in terms of sensory matching, saliency-incongruent and low-saliency are the 2 closest conditions in difficulty. There were no differences of response time between the conditions (Fig. 2*B*).

**Table 2:** *P*-values for pairwise comparisons within each ROI following the repeated measures ANOVA on the area under the curve from the selected ROI

Region	Comparison	*t*	Mean diff	95% CI	*P*
hIP7	SI–SC	2.08	0.28	(−0.63; 0.06)	0.12
	SI–LS	2.58	0.41	(0.01; 0.81)	**0.04**
	SC–LS	0.81	0.13	(−0.26; 0.52)	0.70
hIP8	SI–SC	4.21	0.5	(−0.81; −0.20)	**0.00**
	SI–LS	4.1	0.61	(0.23; 0.99)	**0.00**
	SC–LS	0.84	-0.37	(−0.21; 0.42)	0.68
hIP4	SI–SC	2.71	0.34	(−0.65; −0.02)	**0.03**
	SI–LS	3.14	0.41	(0.08; 0.74)	**0.01**
	SC–LS	0.62	0.07	(−0.21; 0.36)	0.80
hIP1	SI–SC	0.34	0.03	(−0.22; 0.17)	0.93
	SI–LS	4.35	0.31	(0.13; 0.49)	**0.00**
	SC–LS	3.86	0.28	(0.099; 0.47)	0.00
hIP3	SI–SC	2.62	0.21	(−0.41; −0.01)	**0.04**
	SI–LS	4.9	0.42	(0.20; 0.65)	**0.00**
	SC–LS	2.19	0.22	(−0.03; 0.46)	0.09
hIP2	SI–SC	0.62	0.05	(−0.26; 0.15)	0.80
	SI–LS	3.93	0.41	(0.14; 0.67)	**0.00**
	SC–LS	3.51	0.36	(0.10; 0.61)	**0.00**
7A	SI–SC	1.64	0.28	(−0.72; 0.15)	0.25
	SI–LS	3.22	0.47	(0.10; 0.85)	**0.01**
	SC–LS	1.1	0.19	(−0.24; 0.62)	0.52
7P	SI–SC	2.63	0.73	(−1.43; −0.03)	**0.04**
	SI–LS	4.56	0.95	(0.43; 1.48)	**0.00**
	SC–LS	0.96	0.23	(−0.36; 0.82)	0.61

*Notes:* Only the ROIs with significant results are listed in this table. Three pairwise comparisons were made between the 3 saliency-conditions: saliency-congruent, saliency-incongruent, low-saliency. *P*-values in bold survived Bonferroni correction. The column “Mean diff” displays the difference between the means of the 2 conditions that are compared. The column “95% CI” displays the 95% confidence interval of the difference of the 2 means. *Abbrevations:* SC = saliency-congruent, SI = saliency-incongruent, and LS = low saliency.

Stimulus array duration were 124 ms (SD [standard deviation] = 0.078) for saliency-congruent, 214 ms (SD = 0.084) for low-saliency and 235 ms (SD = 0.099) for saliency-incongruent. One-way ANOVA between the mean array durations was significant (*F*_2,42_ = 22.74, *P* < 0.000). Pairwise comparisons showed that array duration for saliency-congruent was significantly lower than for saliency-incongruent (*t* = −5.12, *P* = 0.0001) and low-saliency (*t* = −4.59, *P =* 0.0005). Duration did not differ significantly between saliency-incongruent and low-saliency (*t* = −2.5, *P* = 0.062).

#### Behavioral Analysis during the fMRI Experiment

In the fMRI experiment the duration of array plus mask was held constant but the relative duration of the array was adapted based on the data obtained in the behavioral experiment. This manipulation was done in order to achieve similar performance in accuracy between saliency conditions. In the fMRI experiment, there were no differences in accuracy between the 3 conditions (*F*_2,42_ = 2.36, *P >* 0.1; Fig. 2*D*), indicating that difficulty of each condition was successfully matched. A significant effect of condition upon response time was found during the fMRI experiment (*F*_2,42_ = 7.54, *P =* 0.0016; Fig. 2*E*). Reaction times were longer for saliency-incongruent trials (mean = 370 ms, SE = 16 ms) than for saliency-congruent (mean = 350, SE = 15.7 ms; *t =* −3.10, *pcorr =* 0.016) or low-saliency trials (mean = 355 ms, SE = 15.5 ms; *t =* 3.054, *pcorr =* 0.018).

### Analysis of fMRI Data

#### Multivariate ROI Analysis

We trained and tested a linear SVM to discriminate between the activity patterns of the 3 saliency conditions in the selected Julich cytoarchitectonic areas. Mean ROI classification accuracy was calculated per subject and per ROI for each of the comparisons between conditions. We then evaluated whether this differed from chance level (50%) using one-sample *t*-tests (Table 1).

In hIP7, classification accuracy was significantly higher than chance for the discrimination between saliency-incongruent and saliency-congruent trials (*P <* 0.005) and also between saliency-incongruent and low-saliency trials (*P* < 0.005). Activity patterns in hIP7 did not allow to discriminate saliency-congruent from low-saliency trials. The ability to discriminate between saliency-incongruent and saliency-congruent trials and the inability to discriminate saliency-congruent from low-saliency trials is not compatible with an explanation in terms of the presence or absence of a salient stimulus per se (Table 1).

hIP8 showed a somewhat similar pattern although accuracy only reached significance for the discrimination between saliency-incongruent and low-saliency trials. Performance of hIP4 was clearly distinct as it could only discriminate accurately the saliency-congruent from the low-saliency trials. Apart from hIP7, hIP8, and hIP4 very few other areas allowed for accurate discrimination between conditions (Table 1).

To summarize, these findings indicate a clearly distinct pattern between more posterior and more anterior IPS areas: hIP7 and hIP8 can discriminate between saliency-incongruent and the 2 other conditions but not between saliency congruent and low-saliency. In contrast, more anterior hIP regions such as hIP5 and hIP2 could not discriminate between saliency incongruent and any of the other conditions.

#### ROI-Based Univariate Analysis

The accuracy of the classifier provides insight in the information contained in the activity pattern. However it is also informative to examine the difference in overall response amplitude between conditions. A 3 × 12 repeated measures ANOVA was applied on the area under the curve derived from the normalized BOLD response functions of the 3 main saliency conditions in the 12 core cytoarchitectonic regions (Fig. 2). There was a main effect of saliency (*F*_2,42_ = 10.54, *P <* 0.001), no main effect of region (*P >* 0.1), and a significant interaction between saliency and region (Fig. 4; *F*_24,504_ = 3.22, *P <* 0.001). A number of areas showed significant differences in average response amplitude between conditions. To start, hIP7 as well as hIP8 showed a higher response amplitude during saliency-incongruent than during low-saliency trials. In hIP8 the direct comparison between saliency-incongruent and saliency-congruent trials also yielded a significantly stronger response.

In hIP7 and hIP8 no difference was seen between saliency-congruent and low-saliency trials. hIP4, hIP3, and area 7P were also significantly more active during saliency-incongruent trials than saliency-congruent (Table 2).

**Table 3:** Summary of the significant effects obtained in the multivariate and univariate ROI-based analysis

Region	Can discriminate between (MVPA)	Shows a higher amplitude for (ROI-Univariate)
hIP7	SI vs. SC, SI vs. LS	SI > LS
hIP8	SI vs. LS	SI > SC, SI > LS
hIP4	SC vs. LS	SI > SC, SI > LS
hIP1	None	SI > LS, SC > LS
hIP3	None	SI > SC, SI > LS
hIP2	None	SI > LS, SC > LS
7A	None	SI > LS
7P	None	SI > SC, SI > LS

*Notes:* On the first column are only listed the different cytoarchitectonic ROIs with significant corrected results. Second column lists for which classification pair the accuracy was significantly above chance. Last column shows which condition had a higher BOLD amplitude compared with the 2 others.

When we directly compared the differences among the conditions between the different areas, the difference between the saliency-incongruent minus saliency-congruent condition was significantly larger in hIP8 than in hIP2 (*P* = 0.032), hIP5 (*P* = 0.025), PGa (*P =* 0.014), or PGp (*P =* 0.0099). The difference between the saliency-incongruent and the low-saliency condition was significantly larger in 7P than in e than PGa (*P =* 0.038) and PGp (*P =* 0.016). No differences were found between the areas for the contrast saliency-congruent minus low-saliency.

**Figure 3:**
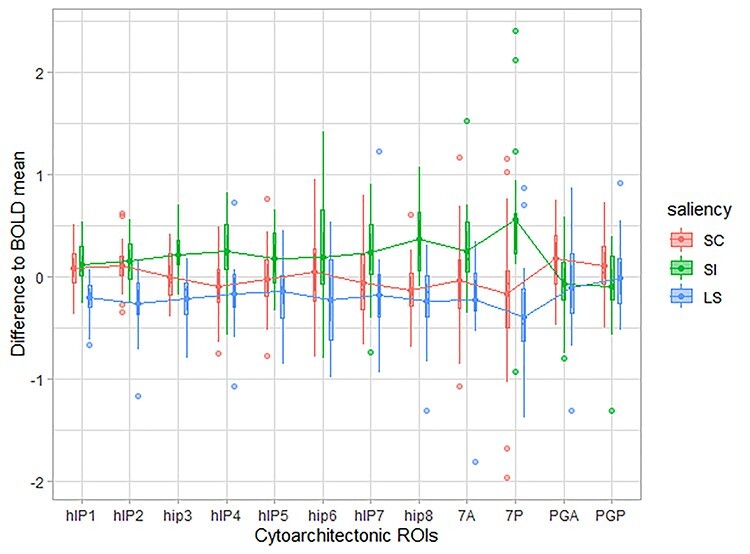
Comparisons of the difference of each saliency-condition to the mean of the BOLD response within each ROI. On the *x*-axis are the 12 cytoarchitectonic regions ordered based on their location in the landmark anatomical regions (inferior parietal lobule, IPS, superior parietal lobule). On the *y*-axis is the difference between the mean area under the curve for a given condition to the mean area under the curve of all-conditions within a specific ROI. Saliency-congruent (SC) is in green, saliency-incongruent (SI) in red, and low-saliency (LS) in black.

In summary, as shown by the interaction plot (Fig. 3), hIP8 shows a significantly higher response during the saliency incongruent condition compared with saliency congruent and compared with low-saliency condition. This univariate pattern in hIP8 is distinct from that seen in most of the other IPS cytoarchitectonic areas except for hIP4 and hIP3, and the SPL area 7P (Table 3).

**Figure 4:**
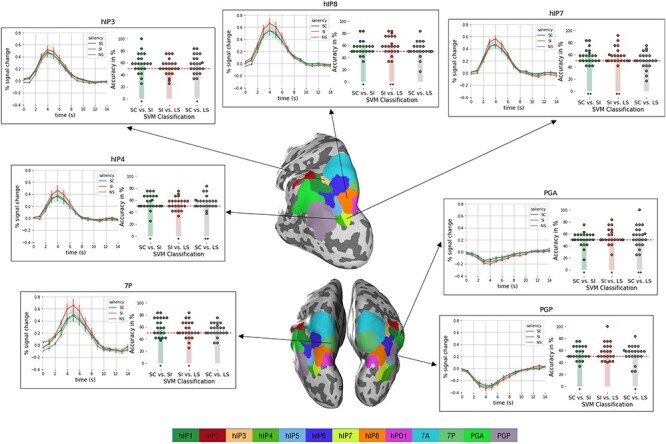
Overview of the univariate and multivariate analysis in the different hIP areas. Seven areas are highlighted here. hIP8, hIP4, hIP3, and 7P were significantly more active in the saliency-incongruent trials compared with the saliency-congruent or low-saliency trials. HIP7, hIP8, and hIP4 could distinguish between saliency-incongruent and saliency-congruent or low-saliency trials. PGa and PGp were able to discriminate between saliency-congruent and low-saliency trials.

**Figure 5:**
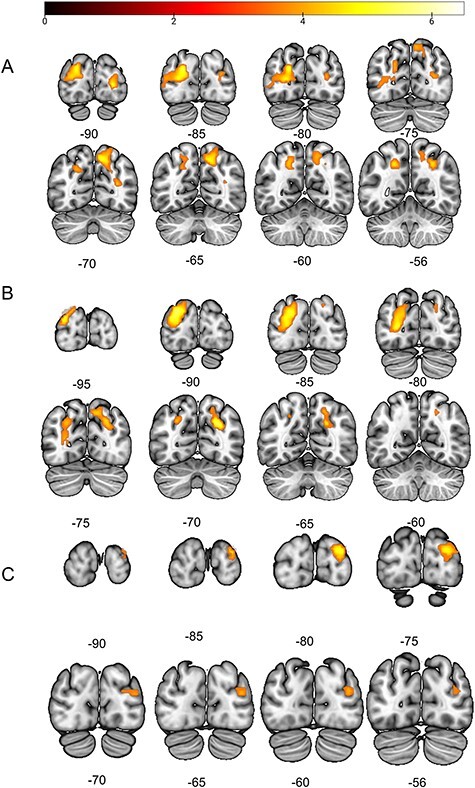
Results of the searchlight classification analysis between saliency-congruent and saliency-incongruent trials, with the relative difference in stimulus array duration and relative difference in RTs controlled as covariates. (*A*) Results of the searchlight classification analysis between saliency-congruent and saliency-incongruent. (*B*) Results of the searchlight classification analysis between saliency-incongruent and low-saliency trials. (*C*) Results of the searchlight classification analysis between saliency-congruent and low-saliency trials. Results are FWE-corrected at *P* < 0.05 at the cluster level. Color bars represent *T*-values of the univariate analysis.

**Figure 6:**
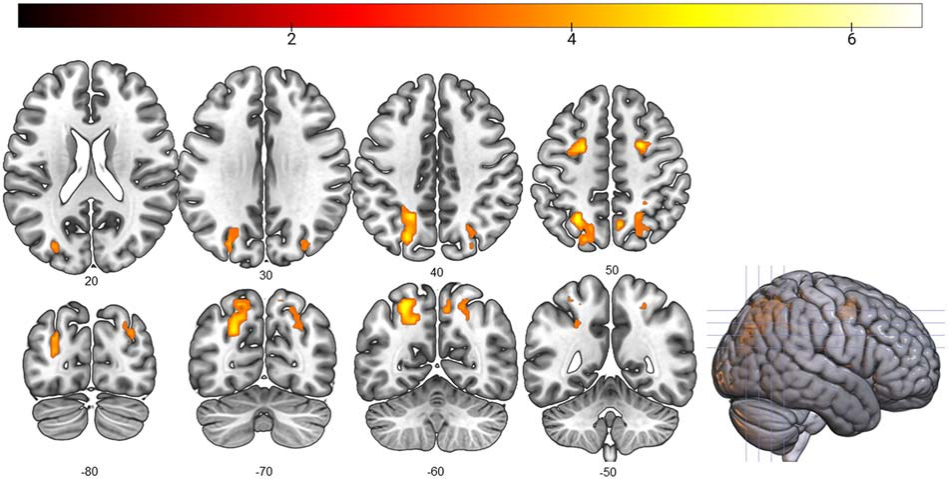
Univariate results for the saliency-incongruent > saliency-congruent contrast. Results are FWE-corrected at *P* < 0.05 at the cluster level. Color bars represent *T*-values of the univariate analysis.

**Figure 7:**
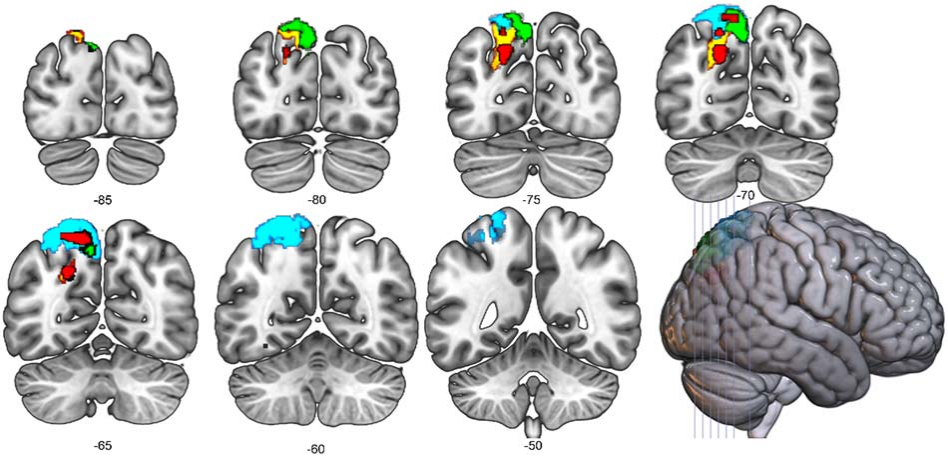
Lesions on the IPS and superior parietal lobule of patient H.H. from Gillebert et al. (2012). In red is the left focal ischemic lesion. The 3 other regions overlayed are the maximum probability maps of hIP8 (orange), 7A (light blue), and 7P (green).

#### Multivariate Whole-Brain Analysis

In order to determine whether other brain regions than our a priori Julich areas were involved during the experiment, we performed a searchlight analysis on the GLM model with the 3 saliency conditions as factor. We trained and tested a searchlight SVM model on the functional images to investigate which regions could discriminate between the 3 saliency-conditions (saliency-congruent, saliency-incongruent, and low-saliency). Classification accuracies between saliency-congruent and saliency-incongruent trials were significantly above chance in the posterior and middle IPS bilaterally, as well as in the inferior temporal gyrus (Fig. 5*A*). Classification accuracy between saliency-incongruent versus low-saliency trials, differed from chance in the left posterior IPS (Fig. 5*B*). For saliency-congruent versus low-saliency trials, activity differed from chance in the right middle IPS at the uncorrected level (*P* = 0.015) but not at the FWE-corrected level (*p*_FWE-corr_ = 0.081; Fig. 5*C*).

Further analyses were done in order to control for the difference in array duration and the difference in RT between conditions. We investigated the neural correlates of these measures by adding the relative difference in array duration and RTs as a covariates in our multivariate searchlight analysis. Relative differences per subject were regressed out during the second level SPM analysis. For each of 3 classifications (SC vs., SI, SI vs., LS, and SC vs., LS), we compared the results of the analysis with and without the covariates, and found no significant differences. Only the analyses that include the covariates are reported in this study.

#### Univariate Whole-Brain Analysis

The saliency-incongruent > saliency-congruent contrast showed a bilateral activation in the IPS as well as in the upper premotor cortex (Fig. 6). Although peak activation for the left parietal cluster sat in the middle IPS, the cluster extended from the most posterior part of the posterior IPS to the middle of hIP3. Similarly, to the left hemisphere, the right parietal cluster spanned the entirety of the posterior IPS, extending anteriorly until area hIP3. The reverse contrast did not show significant results.

### Effect of Position

#### Multivariate ROI Analysis

Classification between left and right target pairs did not reach significance when correcting for the 12 multiple comparisons. However, when the threshold was lowered to an uncorrected *P* < 0.05, hIP8 (*t* = 1.8, *P* = 0.018, CI = [23.9; 92]), hIP4 (*t* = 1.02, *P* = 0.024, CI = [29.9; 81.5]), PGa (*t* = 1.18, *P* = 0.025, CI = [27.1; 77.43]), and hIP2 (*t* = 0.23, *P* = 0.04, CI = [29; 81]), could all differentiate between left and right target pairs.

## Discussion

In this event-related fMRI experiment, we manipulated visual salience and behavioral relevance in a change-detection task and analyzed the results within a cytoarchitectonic reference frame with both uni- and multivariate methods. Previous studies of IPS cytoarchitectonic areas have been based on either univariate analysis (Gillebert et al. 2013) or connectivity analysis (Uddin et al. 2010) or a meta-analyses of prior fMRI studies. According to the a priori hypothesis, the posterior end of the IPS would be mainly sensitive to the presence of salient stimuli and the middle segment of the IPS mainly to the incongruence between the location of the salient stimuli and the endogenous task requirements. Contrary to our hypothesis, activity patterns in the posterior IPS, that is, area hIP7, allowed for accurate discrimination between saliency-incongruent and saliency-congruent trials. Activity patterns in both hIP7 and hIP8 also allowed for accurate discrimination between saliency-incongruent and low-saliency trials, but not between saliency-congruent and low-saliency trials. Response amplitude in hIP8 was higher during saliency incongruent than during saliency congruent trials. Taken together these findings strongly argue against a pure sensory or saliency effect as the explanation for the effects in hIP7 and hIP8. hIP4 showed a clearly different profile: The activity pattern in hIP4 allowed for discrimination between saliency-congruent and low-saliency, a pattern that would be expected in an area that codes for the sensory or saliency effects per se.

In order to equate accuracy between tasks, the array duration was adapted based on the individual’s performance during a prior behavioral session. This procedure succeeded in matching accuracy between the saliency-incongruent, the low-saliency and the easiest, saliency-congruent condition. This required a longer array duration for the former 2 conditions than for the latter condition. In order to equate the total duration of sensory stimulation between conditions, the duration of the mask was adapted accordingly. The stimulus duration was longer for the saliency incongruent and the low-saliency condition than for the saliency congruent condition. It did not differ significantly between the saliency incongruent and the low saliency condition. Since the pattern in hIP7 and hIP8 allowed to discriminate the saliency incongruent from the low saliency condition (which are matched in duration), this effect cannot be attributed to differences in stimulus duration. Furthermore, the activity pattern in hIP7 and hIP8 did not allow to discriminate saliency congruent and low-saliency conditions, which were as different in stimulus duration as saliency congruent and saliency incongruent conditions. This was confirmed by a whole-brain univariate and multivariate analyses where we used stimulus duration and RT as covariates. The discriminative power of patterns of activity in hIP7 and hIP8 for discriminating saliency incongruent from low-saliency and from saliency congruent were still significant even when stimulus duration and reaction times were covaried out.

The current study shows that bottom-up saliency by itself cannot explain the profiles of hIP7 and hIP8. Indeed, bottom-up saliency is the same for both the saliency-congruent and the saliency-incongruent condition. Furthermore, if these areas were involved principally in a first attentional sweep based on saliency, we would expect it to discriminate between low-saliency and saliency-congruent conditions, and not only between low-saliency and saliency-incongruent conditions.

hIP7 and hIP8 coincide with the posterior, descending segment of IPS. Left and right posterior IPS are known to be visually responsive, with an increase in response when attention is oriented towards the contralateral side (Yantis 2001; Vandenberghe et al. 2005, 2012). In Gillebert et al. (2012), a patient who had a focal ischemic lesion in the left IPS was studied (Fig. 7).

The lesion outline of this patient falls within the borders of hIP8 and of areas 7A and 7P. In a spatial cueing paradigm, orientation discrimination was within the normal range when a single stimulus was present either ipsi- or contralesionally. When the contralesional stimulus had been cued invalidly or was presented together with an ipsilesional stimulus, a deficit was present. The current paradigm differs by the absence of a prior cue and the use of a visual search paradigm. Activity in posterior IPS was not so much determined by the presence or absence of salient stimuli but more critically so by a prioritization conflict caused by the presence of salient stimuli at a distance of the locus of endogenous orienting. In the past, we interpreted the activity profile of posterior IPS (IPS0/1) as attentional enhancement of visual responses based on the direction of attention, leftward versus rightward. The current data shed further light on the function of this area. In previous studies, attention was directed to a given location based on a central spatial cue. In the current paradigm, there is no prior cue and attention is oriented based on the features of the stimulus array. The saliency-congruent condition did not differ substantially from the low-saliency condition in hIP7/hIP8, whereas the incongruency between the focus of exogenous orienting and the endogenous focus of attention had a strong effect. hIP4 also demonstrated a difference of activity between the saliency-incongruent condition and the saliency-congruent and low-saliency condition. However, it is the only 1 of the 3 that can successfully discriminate between saliency-congruent trials and low-saliency trials. This may indicate that specific area sits earlier in the attentional processing stream and is more sensitive to the early sweep of bottom-up saliency.

We propose that the profile of hIP7/hIP8 can be accounted for in terms of individuation of attended objects and locations (Xu and Chun 2006). More locations and objects are attended to in the saliency-incongruent than in the saliency-congruent condition. According to Xu et al. (2009b), in a change-detection task, posterior IPS is sensitive to the number of locations (1 vs., 4 locations), regardless of whether the objects occupying these locations are all identical or differ, and regardless of the complexity of the shapes. Middle IPS shows a higher response when all objects differed from each other (1 same object versus 4 different objects). Middle IPS is also involved in VSTM, tracking multiple objects appearing at a same location (Xu and Chun, 2006b). However, based on the current data, we cannot discriminate an object-based mechanism from a location-based mechanism. The conflict between the priority status of the salient and the relevant stimuli may be a critical factor beyond the number of locations/objects attended but that would also require further investigation. Under more natural circumstances and free gaze conditions, the posterior IPS plays a role in the processing of salient stimuli while the supramarginal gyrus is more involved in task-related search (Macaluso and Shih 2018). The exact operation underlying the differential response in hIP7 and hIP8 requires further investigation.

Another cytoarchitectonic area, hIP3, has also been implicated in visuospatial attention (Gillebert et al. 2013): It is more active when participants have to select between stimuli that are simultaneously present. It is also involved in VSTM that resists intervening stimuli and for rejecting distracters with high similarity to the target (Bettencourt and Xu 2015). Xu and Chun (2006) differentiate between the individuation of attended locations and identification. According to Xu (2018), posterior IPS is specialized in the computation of topographic priority maps, whereas middle IPS is more visual working memory based.

Sprague et al. (2018) also manipulated behavioral relevance and saliency in an fMRI experiment. In IPS0 and IPS1 they mainly found an effect of behavioral relevance rather than salience, while earlier areas show a combined effect. They reported that the activation profiles of IPS0 and IPS1 only index the location of relevant object, even in the presence of irrelevant but salient distractor. This can be reconciled with our data if participants orient attention to both the salient locations and the target locations in the saliency-incongruent condition. Under these circumstances, the location of the salient stimuli and of the target locations may both be considered relevant by the participant. This effect could be enhanced by the fact that in some trials the salient stimuli help identify the location of the target stimuli. The difference between the 2 experimental results may relate to the efficiency with which distracters are rejected. In Sprague et al.’s paradigm, subjects are cued for a feature which belongs to only 1 out of the 2 stimuli present, allowing for a faster and more efficient rejection of the irrelevant stimulus. In our case, participants need to find 2 targets amid 6 more distracters. This goes in line with past research showing the IPS’ involvement in multi-target visual detection (Xu, 2007; Praß and de Haan 2019). Our paradigm more closely resembles the one found in Bertleff et al.’s study (Bertleff et al. 2016) where participants have to find a specific target amidst 7 other distracters, one of which is highly salient. In one condition, the location of the target is pre-cued, and in the other condition participants have to search the display for the target (divided attention condition), similarly to our paradigm. Univariate analysis revealed superior parietal activation when comparing the presence versus absence of a salient distracter (cluster peak = *x* = 6 mm, *y* = −56 mm, *z* = 58 mm and *x* = −28 mm, *y* = −60 mm, z = 48 mm). Overall the involvement of IPS and superior parietal lobule in the saliency-present condition is in line with the current report. The current study however also points to the involvement of more posterior IPS areas hIP7 and hIP8.

Area 7P is situated in the medial wall of the superior parietal lobule. This area has been implicated in spatial shifting based on a series of fMRI experiments in humans and in nonhuman primates (Vandenberghe et al. 2001; Molenberghs et al. 2007; Caspari et al. 2015, 2018; Arsenault et al. 2018). Direct comparisons between humans and nonhuman primates using a closely similar behavioral shifting paradigm provides strong evidence that the medial parietal shifting area in humans, that is, 7P, is homologous to area V6a in nonhuman primates. Like hIP4, hIP7 and hIP8, area 7P shows higher activity for saliency-incongruent than congruent or low-saliency trials. This is relatively unsurprising given the number of studies linking area 7P to spatial shifting (Molenberghs et al. 2007; Gillebert et al. 2013).

The activity patterns of PGa were relatively unique in that they allowed discriminating between saliency-congruent and low-saliency trials. The angular gyrus, to which this cytoarchitectonic area belongs, has previously been implicated in the filtering out of distracting stimuli (Shulman et al. 2007). In the low-saliency condition, subjects most likely apply a serial search due to the high target-distracter similarity. In the saliency-congruent condition subjects immediately orient towards the salient stimuli and stimulus identification confirms the target status. Hence, if PGa is involved in distracter filtering one would expect this difference between saliency-congruent and low-saliency condition.

Outside of the cytoarchitectonic areas, the posterior inferior temporal gyrus also differentiated between saliency-congruent and -incongruent. This region overlaps with the lateral occipital complex and plays a role in object identification. Its activation may be related to stimulus identification and error detection when the attentional locus does not contain target object.

The whole brain search, whether univariate or multivariate (searchlight), mainly yielded regions that overlapped with our regions of prior interest. An important note is the absence of an active TPJ in the whole-brain search, whereas one would expect TPJ to be active when salient items were presented outside the endogenous focus of attention. The role of TPJ cannot simply be reduced to exogenous reorienting as this would be seen in the saliency-incongruent condition.

Taken together with findings of prior studies, our findings suggest that hIP7 and hIP8 play an important role in the individuation of attended locations/objects based on saliency as well as endogenous instructions. Together with the lesion data (Gillebert et al. 2011), our data provide converging evidence that areas hIP7–8 are crucial in identifying stimulus locations of salient and of endogenous targets.

Areas hiP7–8 show attentional effects that go beyond attentional enhancement of visual responses to behaviorally relevant stimuli. These areas have a representation of salient stimuli that differentiates between salient stimuli outside versus within the endogenous focus of attention. This is surprising as detection of salient stimuli outside the focus of attention is often related to activity of the TPJ, assumed to be involved in exogenous orienting. Some of our findings are less compatible with the mainstream model of visual attention (Corbetta et al. 2005; Corbetta and Shulman 2011). They are not in line with the concept of a ventral attention network that detects salient stimuli outside the focus of attention. In our paradigm, saliency effects were found much earlier in the attentional processing stream and differentiation between salient stimuli within and outside the endogenous focus of attention were found much earlier in occipitoparietal processing stream. There was no separate area at a distance that was involved. Of course, TPJ may be involved under different circumstances, for example, target singleton detector or as a circuit breaker (Gillebert et al. 2013; Vossel 2014). If it functions as circuit breaker, then in the absence of a clear spatial expectancy, we may not predict involvement in the current paradigm where there was no prior cue.

## Conclusions

To conclude, we manipulated behavioral relevance and stimulus salience in an fMRI change-detection task and analyzed the results using a cytoarchitectonic reference frame. Salient stimuli occurring outside the endogenous focus of attention had an effect in posterior regions of the IPS, mainly hIP8, and hIP7, but not when they occurred within the endogenous focus of attention. This indicates that relatively early areas of the IPS are not only subjected to simple bottom-up sweep of salient objects. These findings are in line with the idea that posterior IPS is involved in the individuation of attended locations/objects, not only based on saliency but on endogenous top-down processes as well. Although this conflict between exogenous and endogenous goals is ongoing as early as hIP8/hIP7, more anterior cytoarchitectonic regions such as hIP3 are related to the identification of stimuli. More research is needed to understand if the effects in hIP8 and hIP4 are purely related to individuation of attended locations, or if they can be explained by the important cognitive demand that one is subjected to when one needs to redirect attentional resources to locations that are not automatically prompted.

## Supplementary Material

Cytosaliency_Supplementary_CC_bhab299

## Notes

We would like to thank all the students who participated in the study. The authors are grateful to the staff of Neurology and Radiology of the UZ Leuven for their help in the data collection, and to Prof. Celine Gillebert for her feedback on the work. No funders had a role in the design of the study, data collection, analysis or interpretation of the data, or in writing the manuscript.
