# Supplementary material for: Posterior Intraparietal Sulcus Mediates Detection of Salient Stimuli Outside the Endogenous Focus of Attention
Source: Cereb Cortex. 2021 Aug 31;32(7):1455–69. doi: 10.1093/cercor/bhab299 (PMC8971085; doi:10.1093/cercor/bhab299)
Supplement: Cytosaliency_Supplementary_CC_bhab299 [file cytosaliency_supplementary_cc_bhab299.zip › Cytosaliency_Supplementary_CC_bhab299.docx]

# Supplementary tables and figures

| Cytoarchitectonic areas |  |  |  | Left hemisphere | | | |  | |  |  | Right hemisphere | | | |
| --- | --- | --- | --- | --- | --- | --- | --- | --- | --- | --- | --- | --- | --- | --- | --- |
|  | *MNI coordinates* | | | | Voxels  _(27 mm³)_ | | Probability thresh. | | *MNI coordinates* | | | | Voxels  _(27 mm³)_ | | Probability thresh. |
|  | x | y | z |  | |  | | x | | y | z |  | |  | |
| hIP1 | -36 | -50 | 36 | 31 | | 60% | | 42 | | -47 | 39 | 56 | | 50% | |
| hIP2 | -50 | -40 | 43 | 63 | | 40% | | 45 | | 39 | 48 | 48 | | 40% | |
| hIP3 | -27 | -57 | 46 | 39 | | 50% | | 37 | | -47 | 52 | 58 | | 50% | |
| hIP4 | -30 | -84 | 25 | 36 | | 50% | | 36 | | -77 | 25 | 55 | | 40% | |
| hIP5 | -26 | -78 | 43 | 37 | | 60% | | 36 | | -65 | 28 | 43 | | 50% | |
| hIP6 | -35 | -66 | 45 | 53 | | 50% | | 39 | | -64 | 47 | 32 | | 60% | |
| hIP7 | -24 | -81 | 25 | 35 | | 40% | | 28 | | -79 | 28 | 52 | | 50% | |
| hIP8 | -19 | -71 | 43 | 52 | | 50% | | 24 | | -68 | 39 | 40 | | 50% | |
| hPO1 | -14 | -80 | 35 | 29 | | 60% | | 25 | | -83 | 43 | 33 | | 60% | |
| 7A | -22 | -63 | 66 | 33 | | 80% | | 24 | | -61 | 65 | 48 | | 70% | |
| 7P | -6 | -78 | 52 | 33 | | 50% | | 12 | | -73 | 59 | 40 | | 60% | |
| PGA | -52 | 60 | 37 | 64 | | 50% | | 62 | | -54 | 25 | 35 | | 70% | |
| PGP | -49 | -75 | 31 | 53 | | 70% | | 49 | | -71 | 34 | 70 | | 70% | |

Supplementary Table 1. Probability threshold and numbers of voxel for each of the ROIs extracted from the cytoarchitectonic areas. The probability threshold was adapted in increment of 10%, until a minimum volume of 27 voxels (of 3x3x3 mm³) was reached in any given ROI.


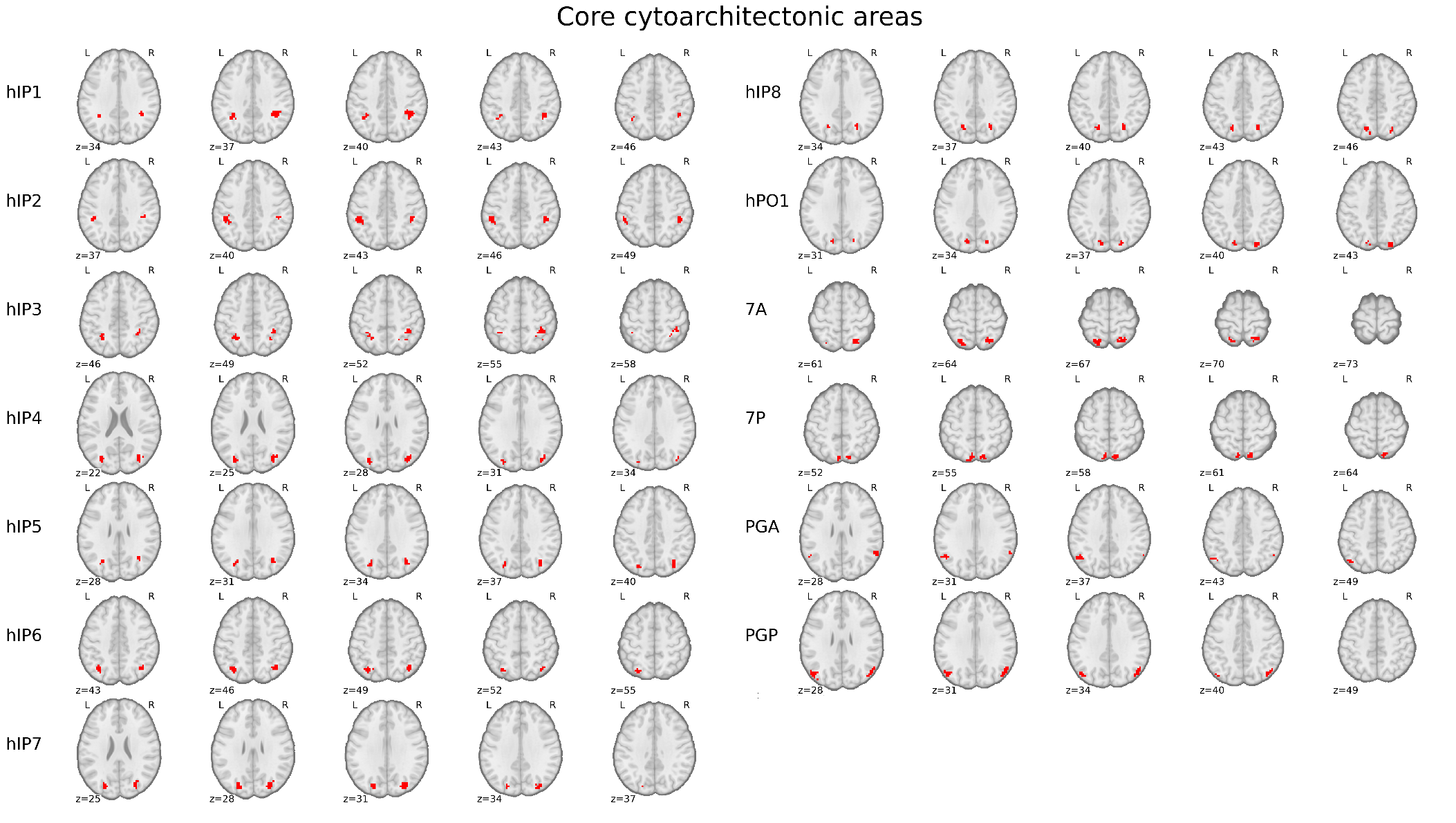


Supplementary Figure 1. ROIs were extracted from the probabilistic brain atlas (Eickhoff et al., 2005). Represented in red are the core cytoarchitectonic areas selected according to the inclusion criteria from Gillebert et al., 2012 (see table. 1 for probability threshold and number of voxel per ROI).

| hIP5 | SI vs SC | 53.4 | (47.5; 59.2) | 0.14 |
| --- | --- | --- | --- | --- |
|  | SI vs LS | 53.3 | (47.5; 59.2) | 0.077 |
|  | SC vs LS | 55.7 | (49.9; 61.6) | *0.005* |
| hIP6 | SI vs SC | 53 | (47.2; 58.9) | 0.16 |
|  | SI vs LS | 56.1 | (50.2; 61.9) | *0.032* |
|  | SC vs LS | 55.9 | (50.1; 61.8) | *0.036* |
| hIP1 | SI vs SC | 55 | (49.2; 60.9) | *0.037* |
|  | SI vs LS | 54 | (48.1; 59.9) | 0.100 |
|  | SC vs LS | 52.7 | (46.8; 58.6) | 0.079 |
| hIP3 | SI vs SC | 58.7 | (52.8; 64.6) | *0.011* |
|  | SI vs LS | 52.1 | (46.2; 58) | 0.237 |
|  | SC vs LS | 57.3 | (51.4; 63.1) | *0.016* |
| hIP2 | SI vs SC | 53.4 | (47.5; 59.2) | 0.13 |
|  | SI vs LS | 51.9 | (46; 57.7) | 0.270 |
|  | SC vs LS | 54.8 | (49; 60.7) | 0.066 |
| 7A | SI vs SC | 57.8 | (51.9; 63.7) | 0.35 |
|  | SI vs LS | 55.8 | (49.9; 61.7) | *0.049* |
|  | SC vs LS | 59.5 | (53.6; 65.3) | 0.098 |
| 7P | SI vs SC | 58.8 | (52.9; 64.7) | *0.020* |
|  | SI vs LS | 54.5 | (48.7; 60.41 | *0.013* |
|  | SC vs LS | 54.7 | (48.9; 60.6) | 0.062 |
| PGP | SI vs SC | 55.3 | (49.4; 61.2) | *0.006* |
|  | SI vs LS | 57.5 | (51.6; 63.4) | 0.074 |
|  | SC vs LS | 54.6 | (48.7; 60.5) | *0.024* |

Supplementary Table 2. Additional P-values of the one-sample t-test on the accuracy of the SVM classifier between the three different saliency-conditions: saliency-congruent vs. saliency-incongruent, saliency-incongruent vs. low-saliency , saliency-congruent vs. low-saliency. Italic p-values indicate significant results that did not survive the multiple comparison corrections. Abbrevations: vs = versus, SC = saliency-congruent, SI = saliency-incongruent, LS = low saliency.

Whole-brain multivariate:

| **Classification accuracy** Saliency-congruent vs Saliency-incongruent | | | | | | | | |
| --- | --- | --- | --- | --- | --- | --- | --- | --- |
|  | *MNI Coordinates* | | |  | | | | |
| Location | x | y | z | Accuracy | kE | Z | *p*_FWE-corr_ |  |
| L posterior IPS | -21 | -81 | 25 | 52.15 | 429 | 4.13 | <0.0001 |  |
| R middle IPS | 39 | -48 | 49 | 52.09 | 436 | 4.13 | <0.0001 |  |
| L Middle IPS | 33 | -6 | 46 | 52.15 | 251 | 3.91 | 0.003 |  |
| R Posterior IPS | 24 | -93 | 4 | 51.95 | 156 | 4.08 | 0.02 |  |

Supplementary Table 3. Results of the searchlight classification analysis between saliency-congruent and saliency-incongruent trials. Significance was set at uncorrected p<0.001 at the voxel level combined with FWE-corrected p<0.05 at the cluster level. The xyz coordinates correspond to the peak of each cluster. kE shows the number of suprathreshold voxels within each clusters.

| **Classification accuracy** Saliency-incongruent vs Low-saliency | | | | | | | | |
| --- | --- | --- | --- | --- | --- | --- | --- | --- |
|  | *MNI coordinates* | | |  | | | | |
| Location | x | y | z | Accuracy | kE | Z | *p*_FWE-corr_ |  |
| L Posterior IPS | -27 | -93 | 25 | 52.12 | 394 | 4.31 | <0.0001 |  |
| R PosteriorI IPS | 27 | -69 | 31 | 51.95 | 186 | 4.26 | 0.009 |  |

Supplementary Table 4. Results of the searchlight classification analysis between saliency-incongruent and low-saliency trials. Significance was set at uncorrected p<0.001 at the voxel level combined with FWE-corrected p<0.05 at the cluster level. The xyz coordinates correspond to the peak of each cluster. kE shows the number of suprathreshold voxels within each clusters.

| **Classification accuracy** Saliency-congruent vs. Low-saliency | | | | | | | | |
| --- | --- | --- | --- | --- | --- | --- | --- | --- |
|  | *MNI coordinates* | | |  | | | | |
| Location | x | y | z | Accuracy | kE | Z | *p*_FWE-corr_ |  |
| R middle IPS | 27 | -96 | 49 | 52.22 | 93 | 5.3 | 0.081 |  |

Supplementary Table 5. Results of the searchlight classification analysis between saliency-congruent and low-saliency trials. Significance was set at uncorrected p<0.001 at the voxel level combined with FWE-corrected p<0.05 at the cluster level. The xyz coordinates correspond to the peak of each cluster. kE shows the number of suprathreshold voxels within each clusters.

Supplementary Whole-brain Univariate

The saliency-incongruent > low-saliency contrast showed a distributed set of significant clusters related to endogenous and exogenous control (table 5). These regions included the middle IPS bilaterally, the left posterior IPS, the right inferior and the superior frontal gyrus, the insular gyrus and some parts of the cerebellum (Vermis 6 & Crus 1). The contrast all conditions > baseline (fig2) showed a very large cluster (kE = 147230) extending dorsally from the cerebellum to the frontal lobe. This cluster encompassed regions like the lateral occipital cortex and the lateral superior occipital gyrus. It also contained regions IPS and FEF from the dorsal attentional network, and reached frontal regions such as postcentral gyrus, middle frontal gyrus and superior frontal gyrus. Furthermore, some subcortical regions (basal ganglia and thalamus) were also included.

| Saliency-incongruent > low-saliency | | | | | *MNI Coordinates* |  |
| --- | --- | --- | --- | --- | --- | --- |
| Location | k_E_ | Z | *p*_FWE-corr_ | x | y | z |
| Superior frontal gyrus (Medial area 8) | 179 | 4.7 | < 0.0001 | 6 | 12 | 52 |
| R Middle Frontal Gyrus (a9/46d, a9/46v) | 51 | 4.27 | 0.006 | 36 | 51 | 19 |
| L Middle IPS  Angular gyrus | 200  55 | 4.19  4.06 | < 0.0001  0.004 | -18  48 | -63  -39 | 52  43 |
| Insular Gyrus | 40 | 4.06 | 0.02 | -36 | -15 | -5 |
| L Middle IPS | 60 | 3.96 | 0.002 | -15 | -45 | 55 |
| L Middle IPS | 271 | 3.96 | < 0.0001 | 15 | -66 | 58 |
| L Posterior IPS | 43 | 3.47 | 0.014 | -15 | -78 | 7 |
|  |  |  |  |  |  |  |

Supplementary Table 6. Univariate results for the saliency-incongruent > low-saliency contrast. Significance was set at the uncorrected p <0.001 at the voxel level combined with FWE-corrected p < 0.05 at cluster-level. kE shows the number of suprathreshold voxels within each clusters.

| Saliency-incongruent > Saliency congruent | | | | | *MNI Coordinates* | | |  |
| --- | --- | --- | --- | --- | --- | --- | --- | --- |
| Location | k_E_ | Z | *p*_FWE-corr_ | x | | y | z | |
| L Upper premotor cortex | 78 | 4.6 | 0.001 | -24 | | -3 | 52 | |
| L Middle IPS | 318 | 4.11 | 0.000 | -21 | | -54 | 40 | |
| R Upper premotor cortex | 53 | 4.29 | 0.006 | 24 | | 3 | 49 | |
| R Middle IPS | 166 | 3.80 | 0.000 | 6 | | -63 | 49 | |

Supplementary Table 7. Univariate results for the saliency-incongruent > saliency-congruent contrast. Significance was set at the uncorrected p <0.001 at the voxel level combined with FWE-corrected p < 0.05 at cluster-level. kE shows the number of suprathreshold voxels within each clusters.


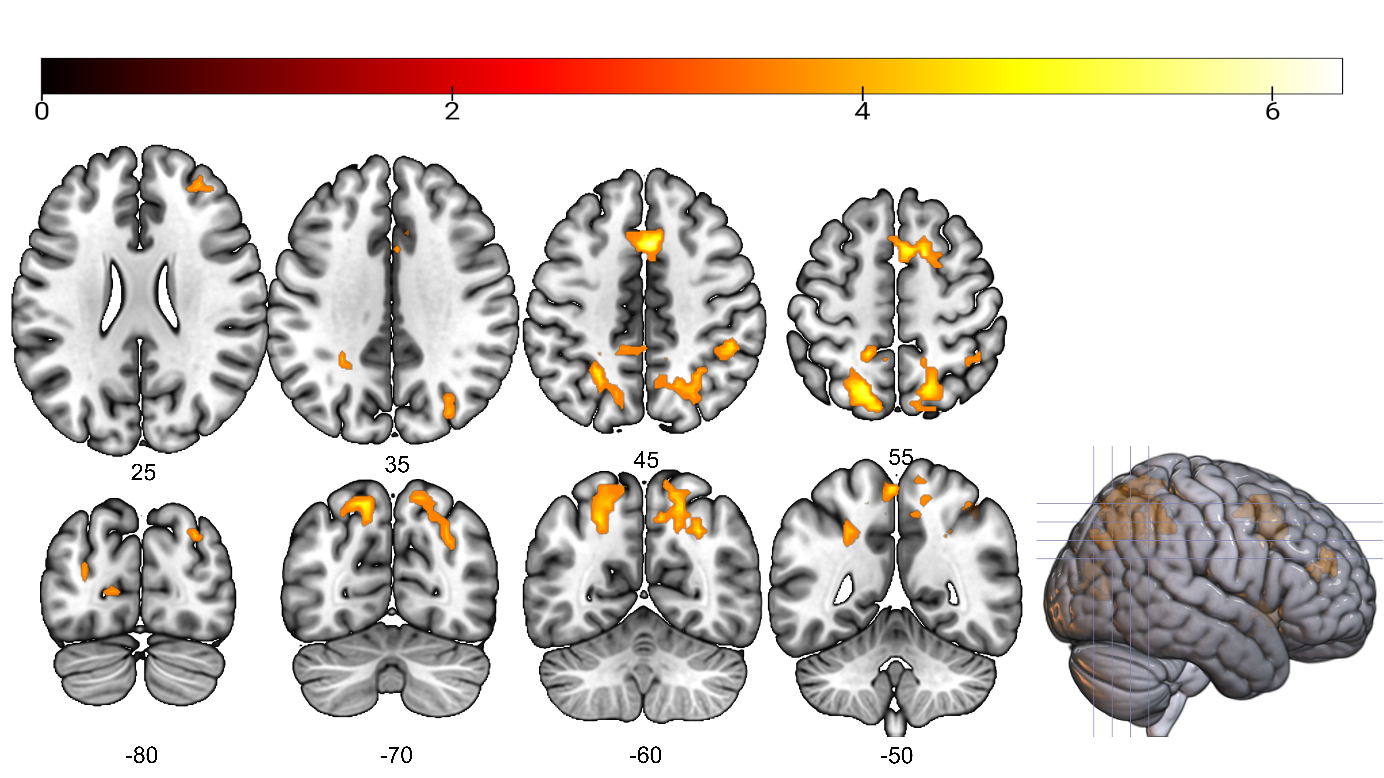


Supplementary Figure 2. Univariate results for the saliency-incongruent > low-saliency contrast. Results are FWE-corrected at p < 0.05 at the cluster level. Color bars represent T-values of the univariate analysis.

| Saliency-congruent > low-saliency | | | | | *MNI Coordinates* | | |  |
| --- | --- | --- | --- | --- | --- | --- | --- | --- |
| Location | k_E_ | Z | *p*_FWE-corr_ | x | | y | z | |
| R Angular Gyrus (Pga/PGp) | 65 | 4.25 | 0.002 | 51 | | -57 | 22 | |
| R Middle IPS | 67 | 4.07 | 0.001 | 39 | | -57 | 40 | |
| R Middle frontal gyrus | 40 | 3.84 | 0.022 | 36 | | 15 | 43 | |

Supplementary Table 8. Univariate results for the saliency-congruent > low-saliency contrast. Significance was set at the uncorrected p <0.001 at the voxel level combined with FWE-corrected p < 0.05 at cluster-level.


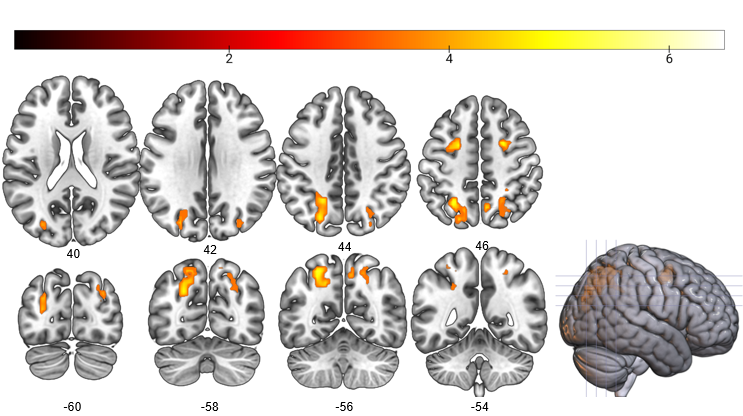


Supplementary Figure 3. Univariate results for the saliency-congruent > low-saliency contrast. Results are FWE-corrected at p < 0.05 at the cluster level. Color bars represent T-values of the univariate analysis.


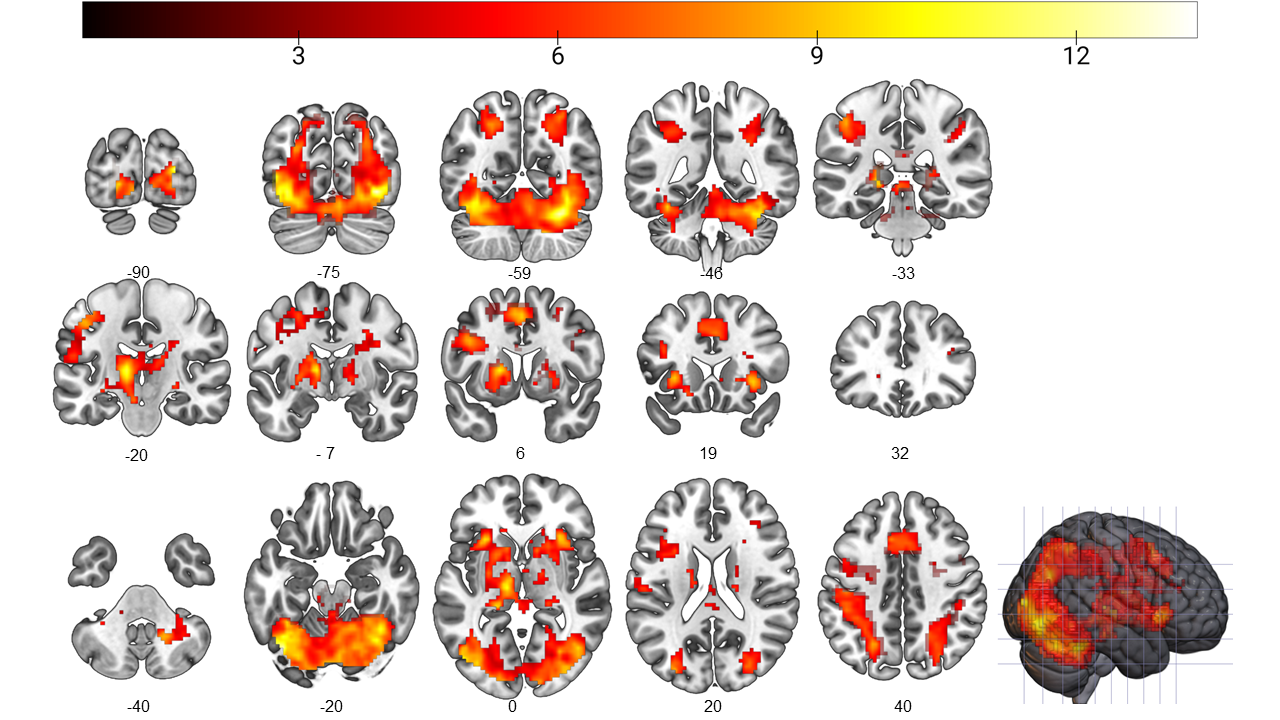


Supplementary Figure 4. Univariate results for the all conditions > baseline contrast. Results are FWE-corrected at p < 0.05 at the cluster level. Color bars represent T-values of the univariate analysis.
